# Sharing Biomedical Data: Strengthening AI Development in Healthcare

**DOI:** 10.3390/healthcare9070827

**Published:** 2021-06-30

**Authors:** Tania Pereira, Joana Morgado, Francisco Silva, Michele M. Pelter, Vasco Rosa Dias, Rita Barros, Cláudia Freitas, Eduardo Negrão, Beatriz Flor de Lima, Miguel Correia da Silva, António J. Madureira, Isabel Ramos, Venceslau Hespanhol, José Luis Costa, António Cunha, Hélder P. Oliveira

**Affiliations:** 1 INESC TEC—Institute for Systems and Computer Engineering, Technology and Science, 4200-465 Porto, Portugal; joana.p.morgado@inesctec.pt (J.M.); francisco.c.silva@inesctec.pt (F.S.); vasco.r.dias@inesctec.pt (V.R.D.); rita.r.barros@inesctec.pt (R.B.); acunha@utad.pt (A.C.); helder.f.oliveira@inesctec.pt (H.P.O.); 2 FCUP—Faculty of Science, University of Porto, 4169-007 Porto, Portugal; 3 Department of Physiological Nursing, School of Nursing, University of California, San Francisco, CA 94143, USA; michele.pelter@ucsf.edu; 4 CHUSJ—Centro Hospitalar e Universitário de São João, 4200-319 Porto, Portugal; claudiaasfreitas@gmail.com (C.F.); eduardo.negrao@gmail.com (E.N.); beatrizflordelima@hotmail.com (B.F.d.L.); miguel.ncds@gmail.com (M.C.d.S.); antonio.madureira@chsj.min-saude.pt (A.J.M.); radiologia.hsj@gmail.com (I.R.); hespanholv@gmail.com (V.H.); 5 FMUP—Faculty of Medicine, University of Porto, 4200-319 Porto, Portugal; jcosta@ipatimup.pt; 6 i3S—Institute for Research and Innovation in Health of the University of Porto, 4200-135 Porto, Portugal; 7 IPATIMUP—Institute of Molecular Pathology and Immunology of the University of Porto, 4200-135 Porto, Portugal; 8 UTAD—University of Trás-os-Montes and Alto Douro, 5001-801 Vila Real, Portugal

**Keywords:** biomedical data, medical imaging, shared data, massive databases, AI-based healthcare solutions

## Abstract

Artificial intelligence (AI)-based solutions have revolutionized our world, using extensive datasets and computational resources to create automatic tools for complex tasks that, until now, have been performed by humans. Massive data is a fundamental aspect of the most powerful AI-based algorithms. However, for AI-based healthcare solutions, there are several socioeconomic, technical/infrastructural, and most importantly, legal restrictions, which limit the large collection and access of biomedical data, especially medical imaging. To overcome this important limitation, several alternative solutions have been suggested, including transfer learning approaches, generation of artificial data, adoption of blockchain technology, and creation of an infrastructure composed of anonymous and abstract data. However, none of these strategies is currently able to completely solve this challenge. The need to build large datasets that can be used to develop healthcare solutions deserves special attention from the scientific community, clinicians, all the healthcare players, engineers, ethicists, legislators, and society in general. This paper offers an overview of the data limitation in medical predictive models; its impact on the development of healthcare solutions; benefits and barriers of sharing data; and finally, suggests future directions to overcome data limitations in the medical field and enable AI to enhance healthcare. This perspective is dedicated to the technical requirements of the learning models, and it explains the limitation that comes from poor and small datasets in the medical domain and the technical options that try or can solve the problem related to the lack of massive healthcare data.

## 1. Introduction

Artificial intelligence (AI) applications are revolutionizing the way we live, creating automatic solutions for several tasks previously performed by humans with fewer errors [1]. The increase in computational power and the amount of data available has opened up the opportunity to develop novel AI solutions in several areas. Image classification has been one of the most successful AI applications, which has allowed, for example, the creation of a self-driving car or facial recognition on social networks [2]. The great innovation for image classification happened with the creation of the ImageNet [3], which is the most recognizable dataset available with a massive amount of labeled data; thus, allowing a technological breakthrough in image classification. Before the creation of ImageNet, there were only relatively small datasets with tens of thousands of labeled images [4]. These image databases only allow simple recognition tasks [4] since all the variability of the population cannot be covered by these small databases. ImageNet is composed of more than 14 million images with 21,841 synsets and more than 1 million bounding box annotations [5]. However, in order to deal with the variability of data, it was necessary to develop architectures with an automatic feature learning capacity. After the creation of ImageNet, several powerful neural network architectures for image classification were developed, such as AlexNet (2012), ZFNet (2013), VGGNet (2014), Googlenet (2014), Inception (2014), ResNet (2015), ResNeXt (2016), DenseNet (2016), Xception (2017), and SENet (2018) [6,7]. Such progress in a few years was possible due to transparency in the evaluation process. A fair comparison with related works must be done to assess real improvements, and the public availability of ImageNet data allowed this evaluation.

An ideal AI-based model should be robust, reliable, and understandable [8]. To achieve this type of model, it is imperative to have an extremely large amount of data that must be representative of all population features. A massive dataset to cover the population’s heterogeneities will only be possible by sharing data collected from multiple institutions. A model trained with this type of representative dataset would then be able to cope with heterogeneities that exist within a population. A robust model should be able to avoid overfitting to generalize well, which would allow the ability to capture the boundaries between classes that will be useful to predict the unseen elements from the test dataset. Generalization is the ability of a classifier to handle new scenarios [9]. One way to test generalization is to evaluate existing models using new independent data that are identically distributed to the original training set [10]. Overfitting occurs when the algorithm, during the learning process, creates a model that performs too well, sometimes by chance, on training data but fails to generalize to new and unseen data (test set) [11]. By allowing the model to achieve an overly specific knowledge of the training examples, it results in a performance decrease on test data [11]. In order to ensure that the model does not rely on the specific elements of the training set, it generally uses strategies such as leave-one-out or splitting a dataset into multiple parts to separate the model training from the validation. With small amounts of data, models will not be able to create a boundary between classes with good accuracy on unseen data and may be biased by some characteristics of the training set [12]. As an alternative way of overcoming the lack of massive training data, Transfer Learning techniques have been explored for several biomedical challenges, allowing to use the knowledge learned with a larger and more generic dataset for a specific application, helping to avoid overfitting by reducing the number of trainable parameters necessary for the learning process of the target task.

Regarding the ideal characteristics of massive data, this has to have five basic dimensions: volume, variety, value, velocity, and veracity, namely the 5 Vs [13]. In fact, the need of large datasets is related with the need to ensure those properties. Only with the extensive collections (high volume of data) is possible to cover the variability of the population using a variety of sources of meaningful data (veracity), which is robust to the noise in the data and labels and allows to capture the statistical relations. The velocity of data generation has an impact on the technological sources available and the protocols, which could change rapidly and influence the acquisition [13]. For example, a novel technological solution can make a traditional clinical exam inadequate and outdated, which makes the previous acquisitions unuseful.

**Figure 1:**
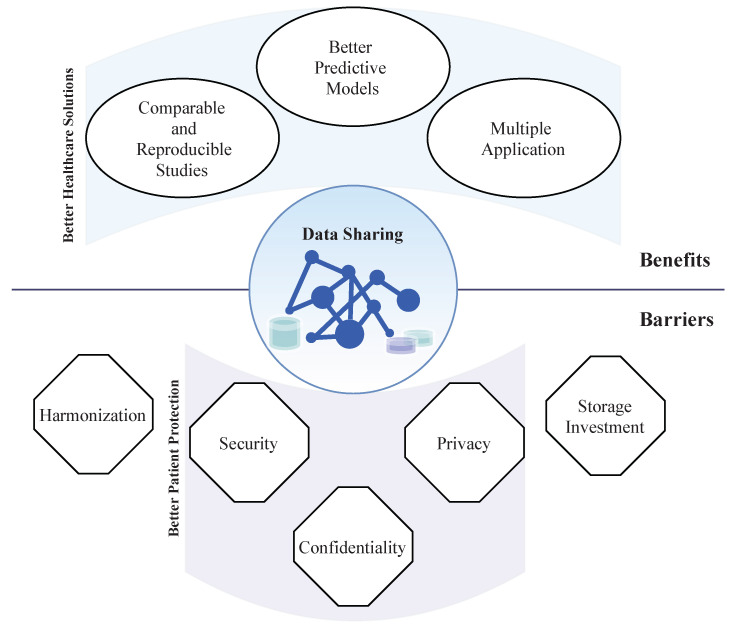
Representation of main benefits and barriers of data sharing. Data sharing has several benefits for healthcare solutions development. Some of the main barriers to data sharing are fundamental for patient data protection.

Almost all technological areas have benefited from the combination of powerful AI-based methods, computational resources, and the amount of data available; however, in healthcare, the situation is different. In the exciting era of data-driven and fast growing AI applications, AI-based development in healthcare has been constrained by the lack of access to large datasets [14]. In the healthcare field, AI-based algorithms can aid in the diagnosis, clinical assessment/staging, screening, and/or treatment plan decision making by providing objective and comprehensive information to clinicians that can be taken into consideration for the final decision [15]. Access to large amounts of biomedical data (medical history, medications, allergies, immunization status, laboratory test results (blood and urine), physiologic signals (ECG, PPG, EEG, arterial pressure), medical image (CT, X-ray, MRI, PET), histopathological images, molecular and all “omics” data) will leverage biomedical knowledge, improving the accuracy of diagnosis, allowing early detection of physiological changes and increasing understanding of the clinicopathological events [16,17,18]. Precision medicine is based on solutions that “provide the right treatments to the right patients at the right time” [19]. Fast and deep characterization of the patient will be aided by AI-based methods that allow the assessment of the main biomarkers that are fundamental for the selection of the most appropriated treatment plan in a short time frame. Massive datasets combined with AI methods allow identifying and inferring meaning of patterns or trends to be directly learned from the data themselves that are not otherwise evident in smaller data sets. Thus, if the hypotheses tested on individual scientific studies were based on a cohort of patients with specific characteristics, chances of lack of generalization for completely different patients are increased. This information will help to develop novel target therapies, innovative biological knowledge, and, as a consequence, personalized medicine. This work gives an overall perspective of the benefits and barriers associated with data sharing (summarized in Figure 1). Current challenges of biomedical data sharing, addressing the impact on the development of healthcare solutions, and major data limitations are discussed.

## 2. Benefits of Data Sharing

Data sharing, which ensures privacy and security for patients, will enable huge and fast progress in healthcare. Currently, automatic methods, such as computer-aided diagnosis (CAD), have promising results and would lead to improvements in patient care workflow and reduce costs related to examinations, the rate of medical interactions and, as a result, the costs [20]. AI applied to medical diagnosis has been shown to achieve, for the detection of some clinical conditions, better performance than clinicians [21]. Nevertheless, there is still a huge potential to improve CAD performance with large amounts of data in several applications.

On the other hand, scientific studies would be comparable and reproducible, which is only possible with shareable datasets. Usually, each scientific study uses a specific and small dataset that is not representative of all the heterogeneities of the population. Consequently, the performance results depend on the samples used and are not comparable across studies. The use of the same datasets would allow comparison between studies to choose the best solution to be implemented in the clinical decision.

With publicly available datasets, it would be possible to disseminate the use of data collection for multiple projects. Biomedical data have the potential to be used for multiple applications to solve distinct problems, or they can be analyzed from different perspectives to extract relevant clinical information.

From this imperative need of data, institutions promote more synergies with other institutions—companies, universities, R&I organizations and hospitals. The need for data has fostered more collaborations to share this resource, which can be considered as a positive side effect in the biomedical field and, ultimately, benefit patients.

## 3. Barriers to Access

The barriers to data sharing for health-related research are complex in nature: a combination of economic and social factors intersect with major legal and ethical concerns, to which can be added relevant technical obstacles to overcome.

In the last years, AI-based solutions have created a fundamental shift in business models, and nowadays, data are recognized as a new currency for companies. Data owners will shape the future by creating the new generation of tech solutions [22,23]. From the biggest tech companies to small start-ups, there is a deep and growing commercial interest in healthcare data, since the future and disruptive clinical technological solutions will depend on the data available for use. Biomedical data are even more valuable than other types of data, given the considerable investment required not only in data acquisition, but also in data preservation and storage.

In the healthcare setting, privacy, confidentiality, and security are the fundamental issues that must be addressed [24,25]. The use of health-related information is often strictly regulated, subject to demanding legal requirements regarding information security and organizational measures (anonymization, pseudonymization techniques and encryption), confidentiality, and the respect for data subjects rights. Furthermore, data protection is essentially a fragmented reality worldwide, often mirroring conflicting or, at least, discrepant conceptions with respect to underlying principles and protected social values. The European General Data Protection Regulation (GDPR) represented a major achievement in this regard. It had a global impact, not only as a result of its extraterritorial provisions but also because it was rapidly acknowledged as a relevant international benchmark in the field of data protection, and it was an important source of inspiration for legislation approved shortly after in several states, from Brazil to California, to the UK in the context of Brexit, or to Canada’s ongoing reforms. However, and despite its uniformization ambitions, the GDPR abounds in vague clauses and open standards, the application of which often requires balancing competing interests. In the case of AI applications, mostly related to biomedical data, the uncertainties are aggravated by the novelty of the technologies, their complexity, and the wide scope of their individual and social effects. GDPR grants to Member States a significant margin of discretion in several fields, where deviations and specifications are allowed: for example, maintaining or introducing further conditions or limitation regarding the processing of biometric, genetic, or health data. Therefore, lack of harmonization persists in this regard, not only internationally, reflecting different cultures and legal traditions, but, to some extent, even within the EEA. Additionally, the regulation of international data transfers represents another relevant barrier. Following the recent and highly debated case Schrems II, judged by the European Court of Justice, data transfers between EU and US blocks was seriously affected [26], jeopardizing health research collaborations. It should be noted that under EU law, mere (remote) access constitutes a form of data transfer for this purpose.

In the technical realm, harmonization difficulties persist. Biomedical data are usually distributed among several heterogeneous and semantically incompatible health information systems, leading to interoperability problems [27]. The goal of data integration is to create a unique semantic reference to ensure data consistency and reuse and, consequently, improve clinical practice, medical research, and personalized medicine [28]. There has been an increase in the adoption of Semantic Web Technologies in healthcare [29]; however, semantic interoperability is far from being applied across all healthcare organizations. Most of these institutions lack comprehensive semantic definitions of the information they contain and have limitations in extracting parameters to solve semantic service discrepancies [30]. In these cases, a human integrator is required to make final semantic decisions [31]. Furthermore, and since biomedical concepts are constantly evolving, the continuous development of semantic integration is essential [31].

Additionally, the majority of medical solutions have been based on supervised algorithms, which require human annotation of the data. There are several annotation platforms, such as MTurk [32] and Figure Eight (formerly Crowdflower) [33], which are usually based on nonspecialized annotators that follow a very restrict and objective set of rules to define the classes for labeling [34]. However, the annotation of biomedical data needs to be done by experts, usually clinicians, due to the complexity of the physiological knowledge required for annotation. In the end, both data collection and annotation represent a large investment for institutions, due to all the issues related to protecting the large number of samples and the human capital involved in AI-based studies [35]. Because of these large investments and the possible opportunities that data can generate, along with legal barriers, data holders have concerns and economic reasons for not sharing the data collections. While, on the one hand, the sharing of such data, either by hospitals, universities, or other entities for public interest or scientific research purposes, is legally possible under certain conditions and undoubtedly represents a high added value for scientific development, the access and control exerted by companies, especially technological and pharma companies, over such databases raises questions as to their ethical use of health data and the possibility of commercial interests taking precedence over scientific impact and the common good [22].

## 4. Possible Solution Strategies

Several initiatives have emerged to help healthcare science improve its ability to develop medical tools and overcome the limitation associated with biomedical data access; however, so far none of them can completely overcome the limitation or represent a solution that will solve the problem in the near future.

### 4.1. Transfer Learning

Recently, TL has been tried as an option to overcome this limitation; however, there is still a lack of large and standardized clinical datasets with potential to be used for multiple biomedical problems [36]. For instance, a dataset from a cohort of patients could be used for several different studies and for the development of multiple AI-based solutions to predict, detect, or assess pathophysiological phenomena, and the multiple associated biological changes. Some studies have attempted to use ImageNet for clinical applications [37,38], using the learning features of a neural network trained on those images; however, due to the dissimilarity between ImageNet and the medical images, its use is limited. Despite the ImageNet creation and the TL approach, there still remains a need for large and standardized clinical datasets that can be used to train models.

### 4.2. Blockchain

Blockchain is an extendable database capable of storing large volumes and various types of biomedical data and is an emerging technology with significant potential in the healthcare domain [39]. Compared to traditional databases, blockchain technology offers many advantages for the biomedical field. Besides its decentralized architecture, the key benefits include immutable audit trail, data provenance, availability, and scalability [40]. It has the potential to address interoperability challenges and has so far been proposed to address several security and privacy issues in a number of different applications in the biomedical sector [41], despite the inevitable tension with important data protection principles and individual rights, when considering the current state of the distributed technology [42]. Although this technology is still more associated with the financial area [43], nowadays, there are many pilot projects currently underway, such as FHIRChain [44], Cancer Gene Trust [45], and Zenome [46]. While the implementation of the blockchain technology in clinical routine can address critical issues related to privacy, legal compliance, avoiding fraud, and improving patient care in cases of remote or emergency monitoring, further production developments, detailed proof-of-concept applications, and research articles are crucial for this technology to move forward and be implemented in the biomedical field. In fact, it must be recognized that despite the immense potential of the supporting architecture of blockchain to transform the delivery of healthcare, medical, clinical, and life sciences, challenges still remain, such as standards and interoperability problems, information privacy and security concerns [40], mainly related to the protection of data flows and data retention periods of datasets. A legal obligation established by the GDPR is to ensure that data subjects can invoke their rights and data-protection principles are implemented by means of appropriate technical and organizational measures.

### 4.3. Synthetic Data

Generative models have recently been applied to generate augmented data for biomedical datasets, thus emerging as a useful tool to increase the number and variability of available examples [47]. Synthetic data are non-reversible, artificially created data that replicate the statistical characteristics and correlations of the original data. This new data overcomes common sharing obstacles, allowing securely access and sharing across institutions, since distributions of real datasets are used to create the synthetic dataset that does not contain identifiable information [48,49], which allows to protect patient privacy while preserving data utility. To build large datasets, synthetic data can be promoted and encouraged, for example, by publishing scientific studies in international journals with the corresponding synthetic data instead of the original datasets. Several studies have been devoted to evaluating synthetic data by analyzing the impact on the performance of learning models compared to the performance obtained with real data. The results showed a small decrease in accuracy for models trained with synthetic data compared to models trained with real data [49]. Synthea™ (MITRE Corporation, Bedford, MA, USA) [50] is one of the most relevant open-source synthetic patient generators due to the massive patient cohort generated; however, the generator showed limited capabilities to model heterogeneous health outcomes [51], and it is still in development. Generating synthetic electronic health records is an enormous challenge because it requires large databases with a combination of linear and nonlinear associations between all medical elements, as well as random associations. Once again, the small databases are the main limitation for data synthesis, restricting the quality of the estimated statistical characteristics of the original data. Furthermore, and since the interactions and correlations are preserved by the synthetic data, the original databases will need to ensure that high-order and complex relationships can be captured. Another limitation is the lack of metrics to evaluate the realism of the generated data. In medical imaging, the validation from clinicians in distinguishing synthetic images from real ones is usually considered the ultimate test, but this evaluation may favor models that concentrate on limited sections of the data (i.e., overfitting, memorizing, or low diversity). Quantitative measures, although less subjective, may not directly correspond to how humans perceive and judge generated images. These, along with other issues such as the variety of probability criteria and the lack of perceptually meaningful image similarity measures, have hindered the evaluation of generative models [52].

### 4.4. International Strategies

In order to overcome the challenges in accessing biomedical data and facilitate its discovery and use, the set of principles proposed by Wilkison et al. [53] should be fulfilled. These requirements are referred to as the FAIR Data Principles and declare that data should be Findable, Accessible, Interoperable and Reusable [54]. More recently, ten principles for data sharing and commercialization have been proposed, which will help guide healthcare institutions to share clinical data with the aim of improving patient care and fostering innovation [55]. The European Commission (EC) has dedicated special attention to this problem and has adopted strategies to boost actions by the European Union (EU), making the importance of personalized medicine a priority through a shared European data infrastructure [56].

In fact, one of the priorities of the Commission for 2025 is the creation of a European Health Data Space in order to promote a better exchange and access to different types of health data, also for health research and health policy making purposes (not only primary but also secondary use of data). Regarding the entire data system, the EC has announced that will be built on transparent foundations and reinforce the portability of health data, as stated in the GDPR [57]. In addition, it will propose a new data governance model and encourage the creation of common European data spaces in crucial sectors. Fully aware of the problem, the EC is proposing a set of measures to increase data availability in the EU to promote the free flow of non-personal data in the Digital Single Market [58] as part of its new data strategy and the underlying data. Indeed, the White Paper on Artificial Intelligence is another pillar of the new digital strategy of the EC, focusing on the need to put data subjects first in the development of technology, in line with the GDPR goals. European GDPR has been in place since May 2018, and it represents a robust data protection guidelines for better quality healthcare [59]. Some of the key privacy and data protection requirements of the GDPR include consent of subjects for data processing, data anonymization, and secure handling the data transfer across borders [59]. The GDPR outlines a special regime for scientific research, demonstrating that research occupies a privileged position within it. In the healthcare sector there is an ethical and scientific imperative to share personal data for research purposes [60].

The most disruptive solution to this major challenge could be the creation of cloud-based repositories of data abstractions (anonymized and abstracted data). The procedures for data abstraction would be developed for each data format using data abstraction techniques such as data masking, pseudonymization, generalization, data swapping, and other techniques using Neural Networks for feature-based masking. These data abstraction procedures would irreversibly convert patient data into anonymized features, preserving the confidentiality and privacy required for biomedical data and thus fully complying with GDPR and all data protection regulations. This type of platform would enable increased reliability of AI applications in the field and provide more training data for AI systems. The data abstractions extracted from medical data would allow the data to be integrated for several other scientific projects as AI-based solutions to improve diagnosis, treatment, and follow-up and contribute to a more precise and personalized medicine. These infrastructures should also address the issue of harmonization of data storage by designing a standard template to define the roles of data that can be submitted in the platform. Since the objective is to collect data from multiple centers, the infrastructure should allow for the submission of the dataset under an objective protocol. All datasets must be checked after the submission, and if they meet all the requirements related to ethical issues and data protocols, they could be added to the repository.

Data from multiple institutions are generated in numerous formats, frequently without a specific structure and with additional semantic information. Data from multiple sources need to be converted into a unified representation, aggregated, and integrated to extract relevant knowledge that can be used by AI-based models to make predictions, exchange data between different healthcare applications, and enable integration with future data [61]. Clinical annotations, medical reports, lab results, imagiological findings, and expressive description of data using standardized procedures are key elements to maximize the quality and applicability of the next generation of the AI-based models [62]. A common infrastructure with common standards for the integration of multiple data sources into one platform (data integration) is fundamental to allow data sharing [63]. Fast Healthcare Interoperability Resources (FHIR) presents standards and technical specifications to define how the information contained in Electronic Health Records (EHRs) should be structured and semantically described [64]. FHIR ensures the requirements for having patient records in an accessible and available format by providing a comprehensive framework and related standards for the exchange, integration, sharing and retrieval of electronic health information. Despite the extreme importance of standards-based data interoperability and EHR integration, their implementation is not consensual [61].

### 4.5. Research Resource for Medical Imaging

Despite all the other technical possible options presented, real data remain indispensable. The Cancer Genome Atlas (TCGA) represents the most important source of data for cancer development from thousands of individuals representing over 30 different types of cancers and containing genomic, epigenomic, transcriptomic, and proteomic data, CT images, histopathological images, etc. [65]. The Cancer Imaging Archive (TCIA) hosts a large archive of medical images of cancer accessible for public download, containing MRI, CT, digital histopathology, etc, and supporting image-related data such as patient outcomes, treatment details, genomics, and expert analyses [66]. Database resources of the National Center for Biotechnology Information comprise hundreds of thousands of databases with biological and genomic information for multiple organisms [67]. Those databases have been allowing the most important developments of AI-based solutions on “omics” and cancer; however, the applications based on medical images still suffer from the insufficient number of cases to use the most powerful deep learning methods. *Stanford Center for Artificial Intelligence in Medicine & Imaging* announced the creation of “Medical ImageNet”, which would be a searchable repository of annotated de-identified clinical (radiology and pathology) images, linked to other clinical information, for use in computer vision systems [68]. Ideally, these databases should comprise data from multiple centers to cover the heterogeneities of the population. The development of these repositories would be more efficient and reliable if the computer systems in clinics and hospitals had built-in software that automatically integrated the annotations and de-identified images into the databases.

## 5. Summary

A large amount of data that include population heterogeneities would allow a revolution in the healthcare field. However, current limitations on access to biomedical data are hindering the development of powerful AI-based tools and restricting improvements in healthcare solutions and medical science development. Recognizing that confidentiality and privacy must be fundamental requirements, it is imperative to find a solution to this limitation. This topic deserves special attention from the entire community working in the biomedical engineering field and key healthcare stakeholders. A paradigm shift in the perception of data in society and institutions, regarding its importance and risks, will create novel solutions to share this extremely valuable resource and perhaps even shift perspectives from patient rights to a duty as a citizen [69].

The limitations imposed on data sharing have hindered a wide range of infectious diseases researchers’ access to crucial data, even at times like the present, during the COVID-19 pandemic. However, during this pandemic situation, the urgent need allowed for exceptional effort and cooperation to develop rapid knowledge and find solutions [70]. Establishing protocols, regulations, methodologies, and definitions to preserve the confidentiality and privacy of data that permit sharing will enable a faster and better scientific response to a similar situation in the future. In fact, the COVID-19 pandemic exposed the extreme need to cooperate, combine effort and share data and knowledge to enable scientific development at a pace never before experienced.
